# Effect of calcium treatment on the browning of harvested eggplant fruits and its relation to the metabolisms of reactive oxygen species (ROS) and phenolics

**DOI:** 10.1002/fsn3.2517

**Published:** 2021-08-13

**Authors:** Qiuyan Ban, Tongjin Liu, Kun Ning, Junjun Fan, Qunxiang Cui, Yanle Guo, Xueming Zai

**Affiliations:** ^1^ College of Horticulture Jinling Institute of Technology Nanjing China

**Keywords:** browning, calcium, eggplant, phenolic metabolism, reactive oxygen species (ROS), ROS metabolism

## Abstract

Eggplant is a popular vegetable in Asia; however, it has a short storage life and considerable economic losses have resulted from eggplant browning. Calcium has been reported to play a key role in the postharvest storage of plants. Here, we found that exogenous calcium application could delay eggplant fruit browning and maintain higher storage quality. The increased browning index (BI), relative electrolytic leakage (REL), and water loss were suppressed by calcium treatment during storage. Delayed browning with calcium treatment might result from a higher phenolic level and suppressed the activity of polyphenol oxidase (PPO). Less H_2_O_2_ and O_2_^‐^ but more activated reactive oxygen species (ROS) scavenging enzymes accumulated in calcium‐treated fruits than in H_2_O‐treated fruits. Moreover, the nonenzymatic antioxidant, ascorbic acid (AsA), was accumulated more in calcium‐treated eggplant fruits. Taken together, our data demonstrated that exogenous calcium application delayed eggplant fruit browning by regulating phenol metabolism and enhancing antioxidant systems.

## Graphical abstract

1. Calcium treatment delayed browning of eggplant fruits during storage.2. Calcium treatment maintained ROS scavenging enzyme activities in eggplant fruits during storage.

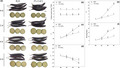

## 1 INTRODUCTION

Eggplant (*Solanum melongena* L.) is a native plant from Southeast Asia that was domesticated more than 4,000 years ago (Das et al., 2011). The world production of eggplant in 2019 was approximately 55.2 million tons, with China being the main producer (http://www.fao.org/). Eggplant fruit is rich in vitamins, dietary fiber, and phytonutrients, especially phenolic compounds, such as caffeic acid and chlorogenic acid, and flavonoids. Eggplant has highly beneficial effects on human health due to its high content of phenolic acids (Salerno et al., 2014; Toppino et al., 2016). These phenolic acids are important due to their various health‐promoting effects (Kaushik et al., 2015).

Fresh eggplant fruits deteriorate rapidly after harvesting and have a very limited shelf life at ambient temperature. In addition, postharvest problems include softening, browning, flavor loss, and disease infections that negatively affect the quality of eggplant fruits during storage or transportation. Many reports have demonstrated that elevated phenolic acid levels in fruit flesh increase the risk of eggplant browning (Taranto et al., 2017). Depending on the mechanism, browning reactions in food products are generally divided into enzymatic and nonenzymatic browning. Enzymatic browning is the main form that occurs during harvesting, transportation, storage, and processing of eggplant fruit (Concellón, CAñón, & RChaves, 2004). Due to tissue damage, phenolic compounds and polyphenol oxidase (PPO) are exposed to oxygen, which triggers the oxidation of phenols into quinones. Subsequently, these quinones and their derivatives polymerize through alternating reactions to form a relatively insoluble brown pigment called melanin (Moon et al., 2020; Taranto et al., 2017). Additionally, changes in antioxidant and nonenzymatic systems have been reported to play a role in the browning of fruits (Hodges et al., 2004; Maioli et al., 2020; Zhang et al., 2015). Therefore, extending the storage life and delaying the decrease in storage quality, especially suppressing browning in eggplant fruits, has become a research hot spot.

As a second messenger, calcium (Ca^2+^) was reported to have a positive function in response to abiotic stresses, including drought, cold, heat, heavy metal, and oxidative stresses (Aldon et al., 2018; Nasir Khan et al., 2009). Recently, postharvest application of calcium was reported to maintain the quality of fresh fruits and vegetables (Li et al., 2020; Xiong et al., 2021). Postharvest application of Ca^2+^ reduced the severity of chilling damage by increasing the calcium in the pulp, thereby delaying browning of the fruit after cold storage (Manganaris et al., 2007). Wang et al. found that exogenous calcium treatment increased cherry firmness and reduced pitting (Wang et al., 2014). 4% Ca^2+^ can improve the postharvest quality and shelf life of bananas, indicating that coating bananas with calcium improves the postharvest quality and shelf life of fruits (Elbagoury et al., 2021). However, little is known about the function of calcium in the eggplant fruit. The objective of this work was, first, to explore the effect of calcium on the browning of eggplant fruit and, second, to investigate the effects of calcium on ROS, phenolics, and antioxidants in eggplant fruits under storage.

## 2 MATERIALS AND METHODS

### 2.1 Fruit materials and treatments

Eggplant fruits (*Solanum melongena* L. cv. “Heilong”) were harvested at a commercially ripe stage (physiologically immature), when the length of the eggplant fruits reached 20 cm, in an orchard in Nanjing, Jiangsu, China. Fruits with uniform size and color and nonvisible damage spots were selected. After removal from the filed heat, the fruits were immersed in 0.05% Tween‐20 solutions containing 0%, 1%, 2%, 3%, and 4% CaCl_2_ for 20 min and then naturally air‐dried for 2 hr at 25°C. In total, 60 eggplant fruits were used for each treatment. All samples were then subjected to room temperature (25°C with 80%–85% relative humidity) storage. Fifteen fruits were sampled randomly on each sampling day.

### 2.2 Browning index detection

Fruit flesh browning was measured as previously described (Kaushik, 2019). The parameters L*, a*, and b* were measured using a Cr‐400 Chroma Meter (Konica Minolta, Japan). The parameters L*, a*, and b* were measured 10 min after the fruit was cut, with 5 fruits per treatment. The value of the browning index (BI) was determined as previously described by using the values of L*, a*, and b* (Palou et al., 1999).

### 2.3 Total calcium content detection

The total calcium content was measured as described previously (Codling et al., 2007; Sun et al., 2020). Over dried fruit tissue (1 g) from 5 fruits was used to determine the calcium content using an Optima 4,300 DV Inductively Coupled Plasma Optical Emission Spectrometer (PerkinElmer) with strontium as an internal standard.

### 2.4 Determinations of storage quality

Fruits from each treatment of 15 fruits per replicate were weighed at each point. For relative electrolytic leakage detection, 15 disks from the pulp tissues of 15 fruits were obtained with a 1 cm‐diameter puncher and incubated in 50 ml ddH_2_O. The electrolytic leakage was first measured at 25°C. Then, the solutions were transported to boiling water for 20 min, and the electrolytic leakage was measured after quick cooling.

After removing the 2 mm‐thick peel, a pressure tester (Effegi Model FT32, Italy) with a 12 mm tapered probe was used to measure the firmness of 15 fruits in each replicate. The maximum force was recorded and expressed in newtons (N).

### 2.5 Determinations of total phenolic and antioxidant metabolite contents

The total phenolic content was detected as previously described (Habibi & Ramezanian, 2017; Shao et al., 2020). One gram of pulp tissue from 5 fruits was ground in liquid nitrogen. Phenolic compounds were extracted in 50 ml of methanol containing 1% (V/V) HCl for 20 min at 4°C in darkness. After centrifugation at 12,000 g at 4°C for 15 min, the absorbance of the supernatant at 280 nm was detected using a spectrophotometer (UV‐1800, MAPADA). Gallic acid was used to construct a standard curve.

H_2_O_2_, O_2_^‐^, CAT, PPO, POD, SOD, and AsA detection assays were performed according to the manufacturer's instructions (Comin).

### 2.6 Statistical analysis

Three biological replicates were performed in each experiment. The experimental data are presented as the means ± standard deviations of three independent replicates. Data were analyzed via analysis of variance (ANOVA), and mean values were compared by Tukey's multiple range test (*p* < .05). All statistical analyses were performed using SPSS18 statistical software package (IBM SPSS Statistics).

## 3 RESULTS

### 3.1 Effect of different calcium concentrations on eggplant under storage

**FIGURE 1:**
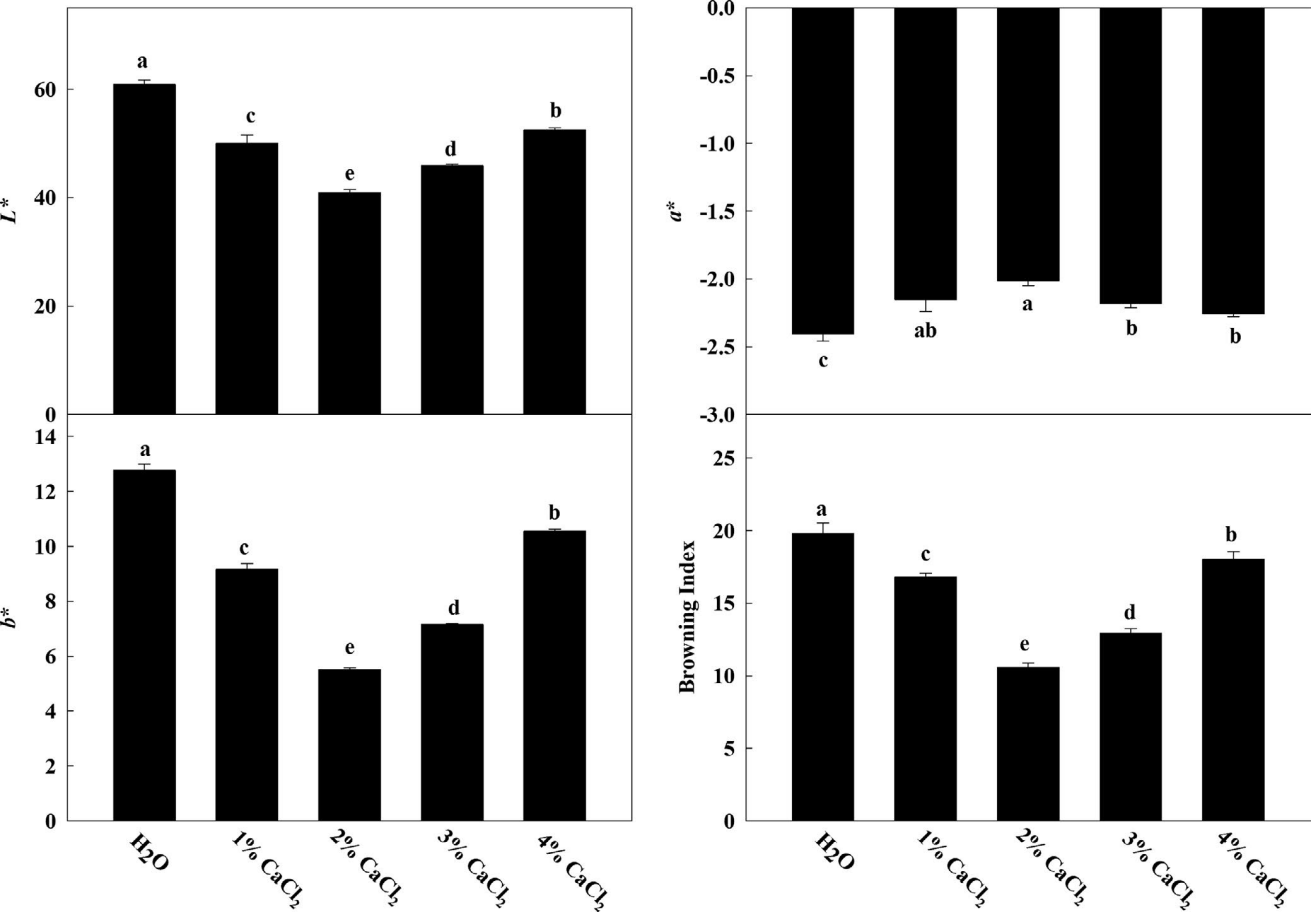
The L*, a*, b*, and browning index (BI) of eggplant fruits with different concentrations of calcium. Data are means of three replicates with *SD*. Different letter indicated significant differences, according to one‐way ANOVA and Tukey's multiple range tests (*p* < .05)

“Heilong” eggplant (*Solanum melongena* L.) fruits were treated with different concentrations of CaCl_2_. As shown in Figure 1, treatment with 1%–3% CaCl_2_ significantly decreased the values of L*, b*, and the browning index (BI) and increased the value of a^*^. As the Ca^2+^ concentration increased (2%–4%), the values of L*, b*, and BI increased (Figure 1). At these concentrations, the effect of 2% Ca^2+^ treatment was the most significant. The BI of 2% Ca^2+^‐treated eggplant fruits was 53.38% that of H_2_O‐treated fruits. However, the BI of 4% Ca^2+^‐treated fruits was not significantly higher than that of H_2_O‐treated fruits.

### 3.2 Phenotype of 2% calcium‐treated fruits and endogenous calcium content

**FIGURE 2:**
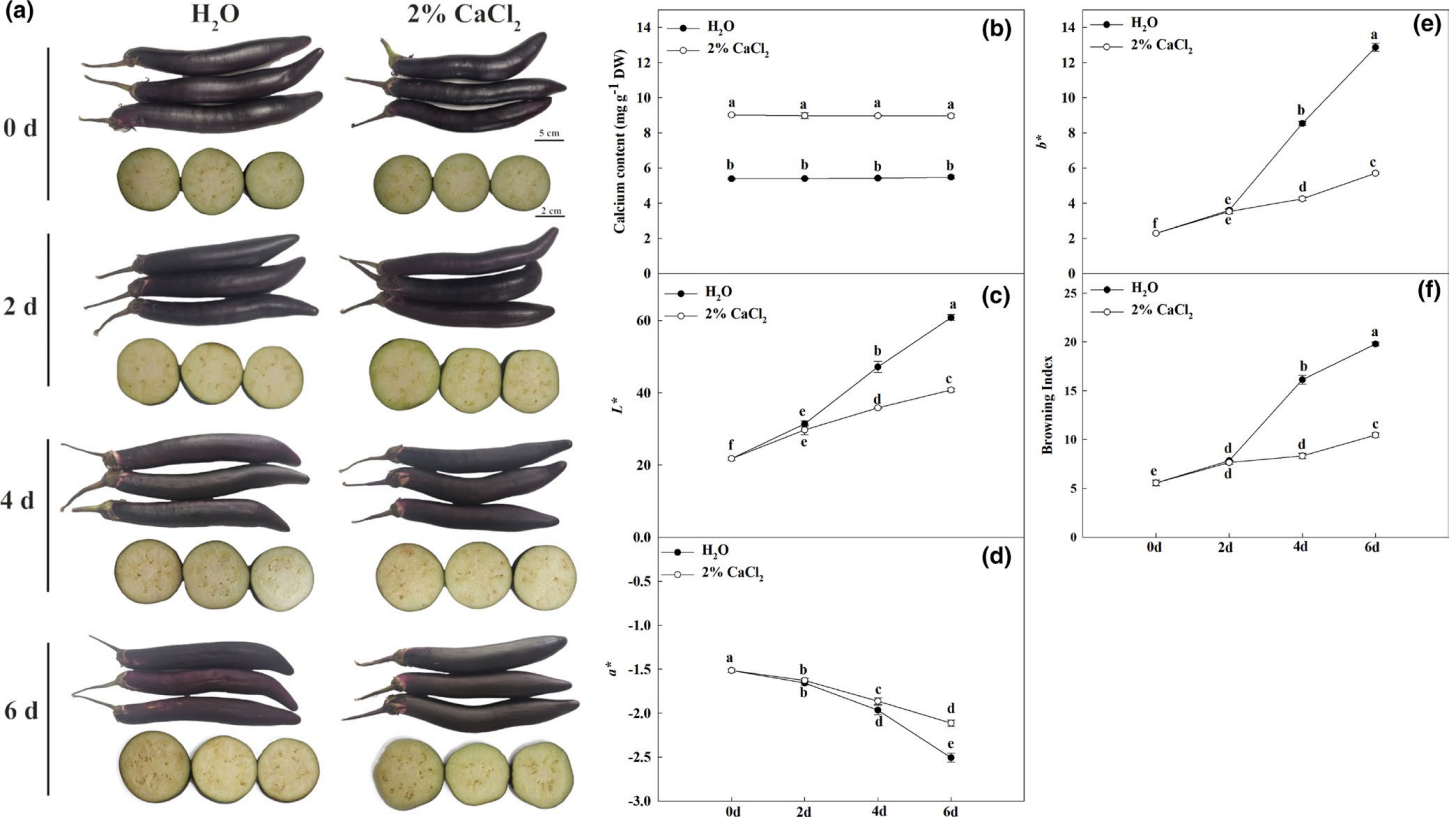
Calcium treatment delayed the browning of eggplant during storage. (a) Phenotypes of H_2_O‐ and calcium‐treated fruits. Upper part: the appearance of H_2_O‐ and calcium‐treated fruits, bar = 5 cm. Lower part: the browning phenotypes of transection of H_2_O‐ and calcium‐treated fruits, bar = 2 cm. (b) The calcium content in H_2_O‐ and calcium‐treated fruits. (c) The L* in H_2_O‐ and calcium‐treated fruits. (d) The a* in H_2_O‐ and calcium‐treated fruits. (e) The b* in H_2_O‐ and calcium‐treated fruits. (f) The BI in H_2_O‐ and calcium‐treated fruits. Data are means of three replicates with *SD*. Asterisks denote statistically significant differences between calcium‐ and H_2_O‐treated fruits (*p* < .05, ANOVA)

Because the effect of the 2% calcium treatment was the most significant among the different calcium concentrations, we chose 2% Ca^2+^ for further investigation. As shown in Figure 2 2% Ca^2+^ significantly delayed the browning and softening of the fruits (Figure 2a). The L*, b*, and BI values of calcium‐treated fruits were significantly lower than those of H_2_O‐treated fruits (Figure 2c,e,f) at 4 and 6 days post‐treatment (dpt). To examine whether exogenous Ca^2+^ application increased the fruit total calcium content, we also detected the total calcium content in eggplant fruits during storage. As shown in Figure 2b, the calcium content in the treated fruits was significantly higher than that in the H_2_O‐treated fruits during storage. The calcium content of treated fruits was more than 65% higher than that of H_2_O‐treated fruits.

### 3.3 Effect of calcium on storage quality in eggplant fruits

**FIGURE 3:**
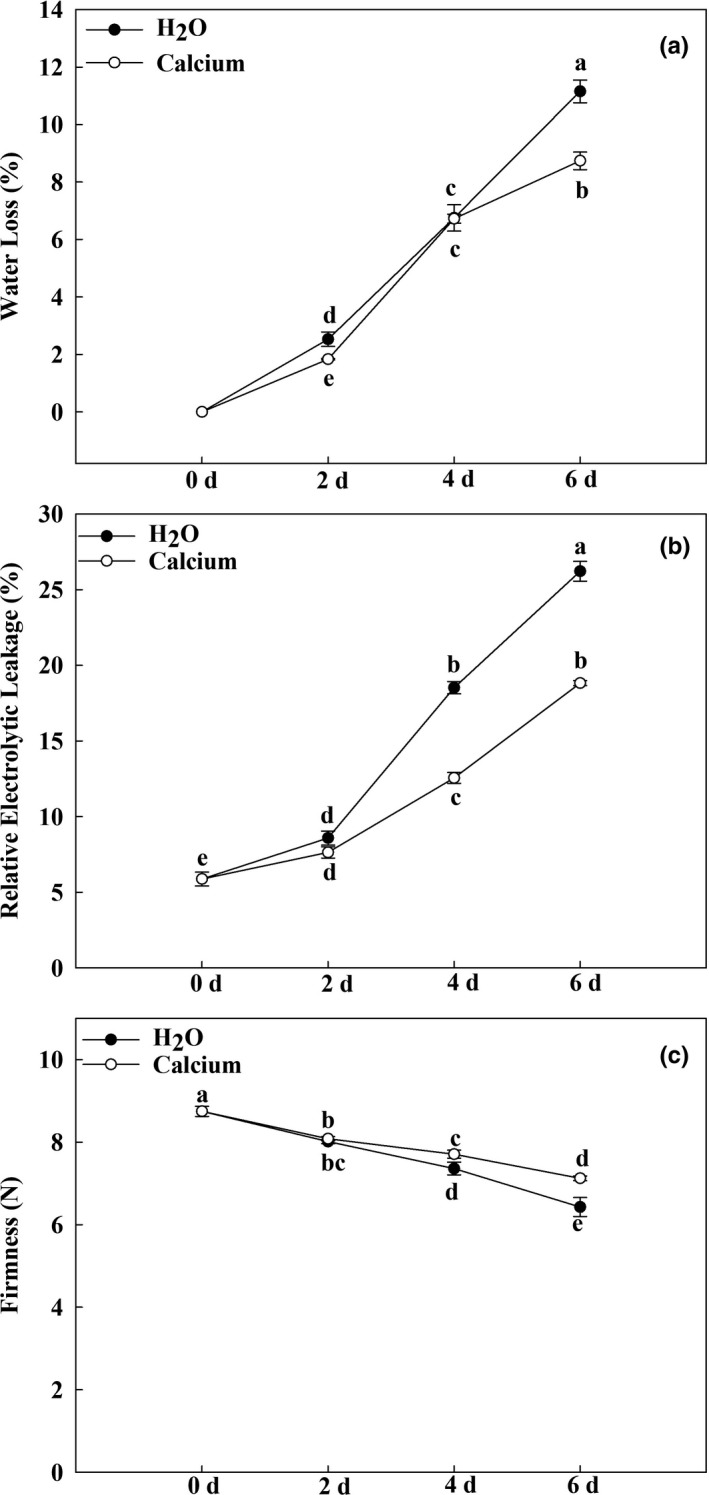
Changes in the storage quality of eggplant fruits during storage. Changes in water loss (a), relative electrolytic leakage (b), and firmness (c) during storage. Data are means of three replicates with *SD*. Different letter indicated significant differences, according to one‐way ANOVA and Tukey's multiple range tests (*p* < .05)

It has been well established that weight loss is an important marker of the storage quality of horticultural products (Gao et al., 2015). Here, our data showed that weight loss in all treated eggplant fruits increased during storage (Figure 3). However, calcium treatment significantly suppressed this increase. The water loss from calcium‐treated fruits was 76.27% that of H_2_O‐treated fruits at 6 dpt (Figure 3). The cell membrane is damaged first during storage. Relative electrolytic leakage (REL) is an important indicator of integrality of the cell membrane. In the present study, the REL gradually increased during storage. However, calcium significantly delayed the increase in REL during storage. At 6 dpt, the REL of H_2_O‐treated fruits was 37.49% higher than that of calcium‐treated fruits (Figure 3). Moreover, calcium treatment delayed the reduction of the firmness of eggplant fruits during storage (Figure 3). These results suggested that calcium treatment maintains the storage quality of eggplant fruits during storage.

### 3.4 Effect of calcium on the activity of PPO and the phenolic content in eggplant fruits

**FIGURE 4:**
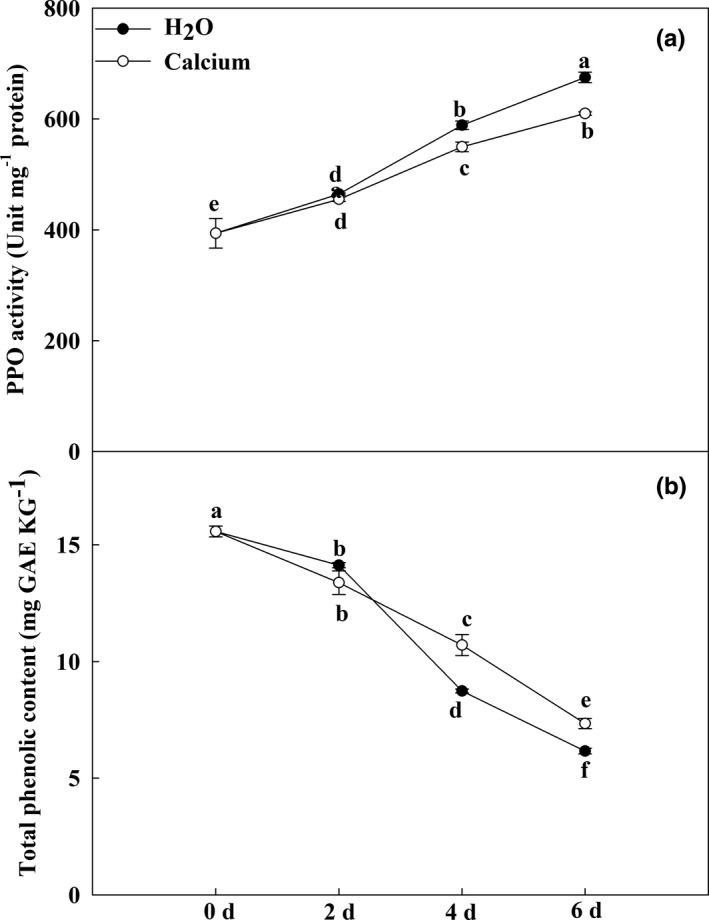
Changes in the total phenolic content and PPO activity during storage. (a) Changes in PPO activity in H_2_O‐ and calcium‐treated fruits during storage. (b) Changes in total phenolic content in H_2_O‐ and calcium‐treated fruits during storage. Data are means of three replicates with *SD*. Different letter indicated significant differences, according to one‐way ANOVA and Tukey's multiple range tests (*p* < .05)

As shown in Figure 2, calcium significantly delayed the browning of fruits. Many studies have reported that PPO and phenolics play an important role in the browning of fruits (Concellón et al., 2004; Maioli et al., 2020). Here, we detected the activity of PPO and the level of total phenolics after calcium treatment during eggplant fruit storage. As shown in Figure 4, the PPO activity of calcium‐treated fruits was lower than that of H_2_O‐treated fruits. Decreased total phenolic contents were detected in all treatments during storage. However, calcium treatment delayed the decrease in total phenolics, especially at 6 dpt (Figure 4). These results indicated that calcium delayed the increase in PPO activity and decrease in total phenolic production, which resulted in a reduction in browning.

### 3.5 Effect of calcium on antioxidant system activity in eggplant fruits

**FIGURE 5:**
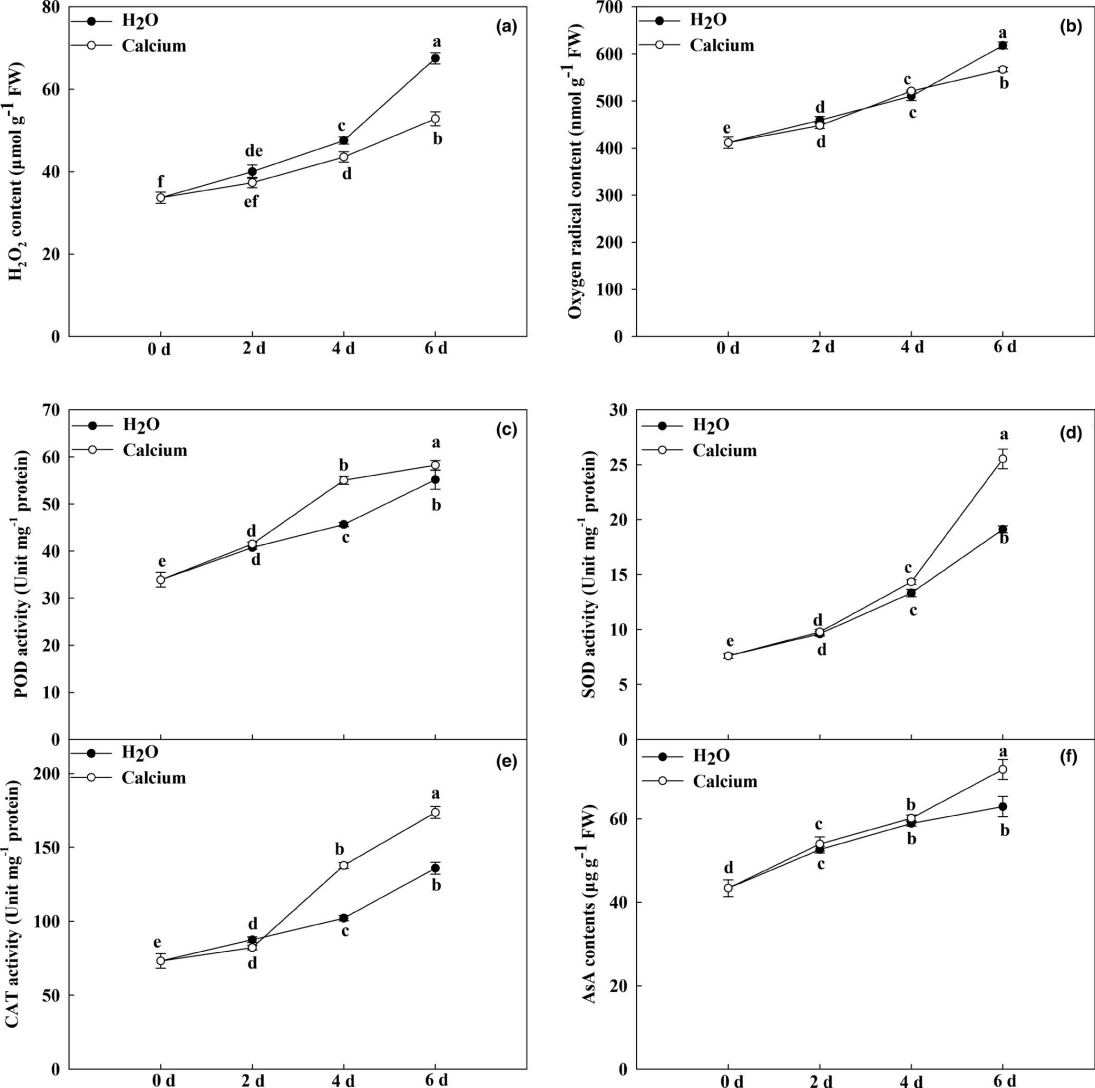
Changes in ROS levels and antioxidant activities during storage. Changes in H_2_O_2_ (a), O_2_^−^ (b), and AsA (f) content during storage. Change in POD (c), SOD (d), and CAT (e) activities during storage. Data are means of three replicates with *SD*. Different letter indicated significant differences, according to one‐way ANOVA and Tukey's multiple range tests (*p* < .05)

ROS scavenging systems play a key role in reducing ROS and stabilizing the cell membrane structure during fruit storage (Hodges et al., 2004; Shao et al., 2020). To analyze the oxidation status during storage, we examined the contents of H_2_O_2_ and O_2_^−^, which are two major stable ROS. As shown in Figure 5, the H_2_O_2_ and O_2_^−^ levels gradually increased during storage. However, calcium treatment obviously delayed this increase. At 6 dpt, the H_2_O_2_ and O_2_^−^ contents of calcium‐treated fruits were significantly lower than those of H_2_O‐treated fruits (Figure 5). We also detected the activities of peroxidase (POD), catalase (CAT), and superoxide dismutase (SOD). After calcium treatment, the activities of POD, CAT, and SOD were significantly higher than those under H_2_O treatment. For example, the activity of CAT in calcium‐treated fruits was 1.28‐fold that in H_2_O‐treated fruits at 6 dpt. In addition, ascorbic acid (AsA) plays a key role in the nonenzymatic antioxidant system (Gallie, 2013; Sun et al., 2018). We also analyzed the change in AsA content during eggplant fruit storage. The AsA level was elevated under all treatments during storage. The AsA content under calcium treatment was significantly higher than that under H_2_O treatment at 6 dpt. These results suggest that calcium treatment improves the ability to produce and maintain higher levels of beneficial antioxidants during storage.

## 4 DISCUSSION

Calcium was established to play a key role in horticultural fruit storage (Elbagoury et al., 2021; X. Kou et al., 2015). However, there are few studies about the effects of calcium application on eggplant fruits. In the present research, we sprayed 0%–4% CaCl_2_ on eggplant fruits to detect the effects of calcium on fruit storage quality. We found that 2% CaCl_2_ application significantly alleviated the browning of eggplant fruits. The calcium‐treated fruits had significantly higher calcium content and lower BI, water loss, and REL values during storage. The lower BI in calcium‐treated fruits may result from a higher phenolic content and lower POD activity. Fewer ROS and enhanced antioxidative activity were detected in the calcium‐treated fruits during storage. These results indicated that exogenous calcium application maintained higher storage quality of eggplant fruits.

Previous studies have shown that an appropriate calcium concentration is beneficial to plant development and adaptation to stress, but excessive calcium application may disrupt the normal metabolism of plants (L. Kou et al., 2014; Sun et al., 2020). A consistent phenotype was observed in our research. The 3%–4% CaCl_2_ treatment showed a decreased effect on the BI of eggplant fruits at 6 dpt (Figure 1), indicating that the effect of exogenous calcium on fruit browning is dose‐dependent. As a secondary messenger, calcium transmits signals received from the cell surface to the cell interior by changing the cytoplasmic concentration, thereby participating in multiple cellular processes, which are decoded by a series of Ca^2+^ sensors (Ranty et al., 2016; Yang & Poovaiah, 2003). Under normal conditions, the intracellular calcium concentration can be well controlled by the mechanism of calcium inflow and outflow in the cell membrane, but a high dose of calcium affects the balance of calcium inflow and outflow, leading to intracellular calcium disorder (Kudla et al., 2010; Steinhorst & Kudla, 2014). These uncontrolled calcium disorders ultimately lead to cell damage.

Phenolics are localized in vacuoles and participate in the browning of eggplant (Holderbaum et al., 2010; Mishra et al., 2012). In a previous study, exogenous calcium alleviated pericarp browning of pears in cold storage (Li et al., 2020). This delayed browning may result from increased endogenous γ‐aminobutyrate (GABA) content, GABA‐related gene expression, and enzyme activity (Li et al., 2020). In addition, calcium treatment reduced the brown spots of pear fruits under cold storage by inhibiting PPO and POD activities and delaying phenolic compound losses (X. Kou et al., 2015). These results indicated that calcium could delay fruit browning by inhibiting PPO activity and phenolic decreases. In our study, the content of phenolics gradually decreased during storage, and calcium treatment significantly delayed this decrease. This suggested that calcium treatment suppressed the decrease in phenolic compounds and fruit browning. This delayed browning may be due to the lower activity of PPO (Figure 4), which could catalyze the phenolic compounds into highly reactive quinones by its oxidizability (González et al., 2019; Plazas et al., 2013).

During fruit storage, ROS were stimulated when the plant cells suffered stress. Extremely high levels of intracellular ROS can damage various components of the cell or activate specific signaling pathways that remove ROS before they can cause cell damage (Asensio et al., 2012). Kou et al. found that the activities of CAT and SOD in exogenous calcium‐treated pear fruits were significantly higher than those in the control treated pear fruits (X. Kou et al., 2015). In fresh fruits and vegetables, the higher activities of enzymes may inhibit the accumulation of ROS, stabilize the cell membrane, and reduce phenolic oxidation by ROS (Li et al., 2020; Moon et al., 2020). Thus, the resulting lower level of ROS may result in delayed browning of pear fruits. Here, less H_2_O_2_ and O_2_^−^ accumulated in the calcium‐treated fruits than in other fruits during storage. Moreover, the activities of the enzymatic ROS scavenging antioxidants CAT, POD, and SOD were significantly higher in calcium‐treated fruits than in H_2_O‐treated fruits. Moreover, the level of the nonenzymatic ROS scavenging antioxidant AsA was also significantly higher in calcium‐treated fruits than in H_2_O‐treated fruits. These results indicated that calcium may elevate the ROS scavenging system to protect cells from oxidative damage.

## 5 CONCLUSION

Exogenous calcium application delayed browning and maintained the quality of eggplant fruits during storage. The lower BI may have resulted from a higher phenolic content and lower POD activity. Higher calcium contents and firmness were detected after calcium treatment. The REL and water loss were suppressed by calcium treatment. Moreover, the calcium‐treated fruits accumulated lower levels of ROS and showed higher SOD, POD, and CAT activities. Additionally, the AsA level was higher in calcium‐treated fruits than in H_2_O‐treated fruits. These results provide further insight into the function of calcium in eggplant fruits storage. Thus, spray application of exogenous calcium onto eggplant fruits can be used to maintain storage quality.

## CONFLICTS OF INTEREST

The authors have no conflicts of interest to declare.

## AUTHOR CONTRIBUTION

**Qiuyan Ban:** Conceptualization (lead); Data curation (lead); Formal analysis (lead); Funding acquisition (lead); Investigation (lead); Software (lead); Writing‐original draft (lead); Writing‐review & editing (lead). **Tongjin Liu:** Data curation (supporting); Formal analysis (supporting). **Kun Ning:** Data curation (supporting); Formal analysis (supporting). **Junjun Fan:** Data curation (supporting); Formal analysis (supporting); Writing‐original draft (supporting). **Qunxiang Cui:** Data curation (supporting); Formal analysis (supporting); Investigation (supporting). **Yanle Guo:** Data curation (supporting); Formal analysis (supporting). **Xueming Zai:** Data curation (supporting); Formal analysis (supporting).

## ETHICAL APPROVAL

Ethics approval was not required for this research.
